# Fatigue-Related Effects in the Process of Task Interruption on Working Memory

**DOI:** 10.3389/fnhum.2021.703422

**Published:** 2021-11-17

**Authors:** Yueyuan Chen, Weining Fang, Beiyuan Guo, Haifeng Bao

**Affiliations:** ^1^State Key Laboratory of Rail Traffic Control and Safety, Beijing Jiaotong University, Beijing, China; ^2^School of Mechanical, Electronic, and Control Engineering, Beijing Jiaotong University, Beijing, China

**Keywords:** task interruption, EEG, fatigue, working memory (WM), 2-back task

## Abstract

Interruption generally has a negative effect on performance by affecting working memory (WM). However, the neural mechanism of interruption has yet to be understood clearly, and previous studies have largely ignored the role of fatigue state. To address these issues, the present study explores the behavioral and electrophysiological effects of interruption on WM performance using electroencephalography (EEG) data. The moderating effect of fatigue is also explored. The participants performed spatial 2-back tasks with math task interruption, suspension interruption, and non-interruption under different fatigue states. The results show that interruption led to increased alpha activity and P300 amplitude, indicating inhibitory control to interference from irrelevant information. Analysis of P200 amplitude revealed that interruption affected attentional reallocation when resuming the primary task. Increased theta power indicated an increased demand for information maintenance during the interruption. A speeding-up effect was discovered after interruption; however, fatigue impaired cognitive ability and further exacerbated the negative effects of interruption on WM and behavioral performance. These findings contribute to a better understanding of cognitive activity during the interruption and of the interaction with fatigue, and provide further support for the theory of memory for goals (MFG).

## Introduction

**FIGURE 1:**
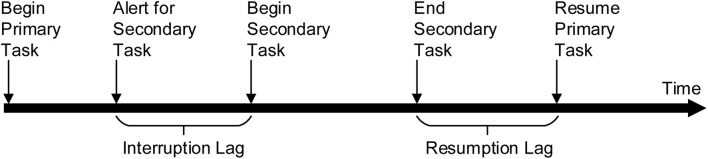
Timeline of the task interruption process proposed by Trafton et al. (2003).

Interruption refers to any event that interferes with work continuity and is not directly related to the main task (Laarni, 2021) where there is an intention to return to complete the original workstream (Boehm-Davis and Remington, 2009). Most studies have shown that interruption leads to a decline in the performance of the primary task, mainly due to resumption lag (Puranik et al., 2020), defined as the time between the end of the secondary task and the recommencement of the primary task (see Figure 1). However, a few studies have argued that interruption has a positive effect on task completion, as despite negatively affecting tasks with higher complexity, it can promote simple tasks (Speier et al., 2003). A key aspect of interruption is how it affects task performance. According to a widely accepted cognitive theory of interruption, memory for goals (MFG), the decline of performance after an interruption is due to decreased memory activation of the primary task based on the working memory (WM) mechanism (Altmann and Trafton, 2002). The interrupted task goals decay during the interruption, and it is necessary to overcome the interference of the interruption task to restore their activation when resuming to the primary task (Monk and Kidd, 2008; Altmann et al., 2014).

Until now, most researchers have adopted experimental methods to reflect the correlation between various factors and interruption through behavioral performance data. Far too little attention has been paid to the brain’s neural mechanism during an interruption. Rejer and Jankowski (2017) studied the interruption effect of advertising pop-ups during reading tasks. By analyzing changes in EEG before, during, and after advertising, they found that interruption reduced frontal/prefrontal beta activity and led to changes in the frontal/prefrontal asymmetry index. Another study on the effect of audio interruption on reading tasks reported that task interruption led to brain activity changes in multiple regions. Frontal lobe, temporal lobe, and sub-lobar activities were most closely related to the interruption (Kalgotra et al., 2019). Clapp et al. (2010) have explored the effects of external interference (including distraction and task interruption) on WM based on a delayed-recognition paradigm. They analyzed the connectivity of the prefrontal cortex (PFC) and visual association cortex (VAC), which are functionally connected WM maintenance of relevant stimuli. Compared to distractions, a significant drop in connectivity during interruption indicates the interrupted WM was not retained but reactivated after interruption. Allocating attention toward interference causes encoded scenes to be “released” during an interruption. The optimal WM relies on effective filtering of irrelevant information through neural inhibition, which prevents limited memory capacity from overloading (Zanto and Gazzaley, 2009). Interruption practice also has a positive effect on WM (Berry et al., 2009). Zickerick et al. (2020b) analyzed the attentional control processes of task interruption and distraction during a continuous number task. The higher P300 amplitude following interruption explained the enhanced attentional reallocation to stimuli, and the decreased frontal theta activity and increased posterior alpha activity indicated reduced attention control resources (Zickerick et al., 2020a). Nonetheless, as Beaton et al. (2013) observed, the current literature on brain activity during interruption is limited, and more research is needed in this field.

Many studies have explored features of tasks that affect post-interruption task performance, such as complexity of the primary task (Speier et al., 2003) and interruption tasks (Cades et al., 2008), interruption frequency (Basoglu et al., 2009), and interruption timing (Bailey and Konstan, 2006). A few studies have investigated the effects of person-related factors, such as age (Monk et al., 2004), gender (Kalgotra et al., 2019). However, no research has been found that surveyed the moderating effect of fatigue on interruption. In many fields, long-term monitoring and operations of human–computer interaction tasks have become the norm, which leads easily to feelings of fatigue. Hence, handling interruptions under fatigue is common and unavoidable. Although Westbrook et al. (2018) considered fatigue factors in interruption in the context of emergency physicians, they studied only the main effects of interruption and fatigue on prescription errors and ignored the interaction effect. Some scholars have studied the workload effect on interruption, finding that interruption has less negative effect on performance when workload is low (Iqbal and Bailey, 2005). There is a close relationship between workload and fatigue, but they are not the same concept (Abe et al., 2009); workload is an objective indicator measured from the perspective of the task, whereas fatigue is the psychophysiological state of the personnel. Therefore, the research results cannot directly explain the effect of fatigue on WM after interruption.

To date, there is no unified evidence on the relationship between interruption and WM-related performance. Physiological research on the interruption process is still in its initial stages. As a critical individual characteristic factor, the effect of fatigue on WM performance after interruption remains unclear. The current study addresses these issues using electroencephalogram (EEG) to measure and record the electric activity of the brain to better understand the effects of interruption on primary task performance and how fatigue affects interruption processes.

Task interruption is related to task switching, WM encoding, maintenance and retrieval, and inhibition of irrelevant information (Zanto and Gazzaley, 2009). The n-back task involves information encoding, updating, and maintenance (Owen et al., 2005). Accordingly, this study uses a spatial 2-back task as the primary task, adding task interruption and suspension interruption. The objective is to explore the electrophysiological mechanism of interruption effects on WM performance by analyzing behavioral data and brain activity during the interruption. We also aim to determine whether and how fatigue affects post-interruption performance by analyzing data differences in fatigue and non-fatigue states.

We focus on theta and alpha oscillations related to WM (Clayton et al., 2015) and Event-related Potentials (ERP) components of P200 and P300 induced by spatial 2-back tasks (Han et al., 2013). Several studies on WM have indicated that different oscillations are related to different control mechanisms in WM (Sauseng et al., 2010). The n-back task has found that decreased alpha band power at parietal electrodes and increased theta power at frontal electrodes accompany an increase in memory load (Zhao et al., 2017). The increase of theta band power is correlated with the demand for episodic long-term and working memory (Klimesch, 1999). A study using the Sternberg paradigm observed that theta power increased in information encoding and was sustained during information maintenance (Raghavachari et al., 2001). Theta oscillations are sensitive to the amount of information, which reflects the memory load (Jensen and Tesche, 2002). In general, alpha activity related to WM load represents information suppression or separation in the posterior parietal region. The activity of the cerebral cortex that represents task-irrelevant information is inhibited to reduce the interference input in the relevant regions and to maintain WM (Manza et al., 2014).

We focused on the P200 and P300 components because these components were previously shown to be evoked by the n-back task (Han et al., 2013). P300 amplitude is sensitive to attentional resources and related to WM processes. P300 may occur when attention resources are active in the frontal area and promote memory operation in the temporal-parietal area (Polich, 2007). In multitasking, attention resources are assigned to different tasks, which reduces P300 amplitude and increases peak latency. Other studies have shown that stimulus switching can also affect P300 amplitude. With an increase in memory load and task processing demand, subsequent attention resource engagement increases P300 amplitude (Kok, 2001). Stimulation with full attention can promote successful memory storage, retrieval, and recognition, and is associated with greater P300 amplitude than divided attention (Curran, 2004). At the same time, P300 is an effective index to evaluate mental fatigue. Research has shown that with the deepening of mental fatigue, the amplitude of P300 decreases significantly (Käthner et al., 2014). Compared with P300, there are relatively few studies on P200. Generally, P200 is distributed around the centro-frontal and the parieto-occipital areas of the scalp (Lenartowicz et al., 2010) and has been considered to reflect the early stage of information processing (Ciecko-Michalska et al., 2012) and is related to effective information selection, attention allocation, and memory recognition (Lenartowicz et al., 2010). P200 amplitude decreases with an increase in attention may suggest greater attention-shifting ability (Wongupparaj et al., 2018).

Based on previous studies, it is necessary to transfer attention from the interruption task, resume the primary task after the interruption, and retrieve and reactivate the primary task’s relevant WM. To explore the detailed cognitive mechanism of the interruption process, provide more physiological evidence and provide theoretical support for interruption management, the hypotheses tested in this study are as follows:

(1) Interruption negatively affects post-interruption performance and increases memory load.
(2) Demand for information inhibition is increased after interruption, resulting in increased alpha power and P300 amplitude.
(3) Cognitive function is impaired in the fatigue state, and P200 and P300 amplitudes after interruption are lower than in the non-fatigue state.

## Materials and Methods

### Participants

We recruited 34 healthy adults (nineteen men and fifteen women) for this study. All were between the ages of 22 and 29 (mean = 23.94, SD = 1.63), right-handed, with normal or corrected-to-normal visual acuity, and had no behavioral or cognitive impairment. Each participant volunteered, provided consent, and was fully informed about the experiment. All participants received $40 in remuneration after the experiment.

### Experimental Design and Stimuli

**FIGURE 2:**
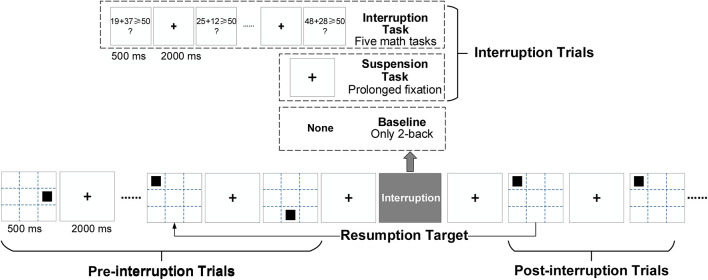
Experimental design based on the spatial 2-back task. There were three types of working memory (WM) tasks. In the interruption task (IT), the interruption consisted of five math tasks. In the suspension task (ST), the primary task (2-back) was suspended by prolonged cross-trial fixation (of the same duration as the math tasks). In the baseline condition, only the 2-back task was performed.

By definition, interruption is caused by a secondary task or an unexpected pause (Puranik et al., 2020). We designed the task shown in Figure 2, taking the spatial 2-back task as the primary task. A black square was shown for 500 ms at one of eight predefined screen locations except for the center. The screen showed a fixed cross in the center for 2,000 ms between the two trials. The identity positions of each stimulus in the spatial task were determined at random to achieve a roughly uniform distribution. Stimuli were presented on a 24-inch monitor (60 Hz refresh rate; 1,920 × 1,200 pixels) placed at 70 cm distance from participants.

Three types of tasks were used: an interruption task (IT), a suspension task (ST), and the baseline condition (Base). The only differences between the tasks were the interruptions. In the IT condition, a math problem requiring addition within 100 was the secondary task. A math task was presented for 500 ms, followed by a fixed cross for 2,000 ms. In the ST condition, the interruption was a suspension with a prolonged presentation of the fixed cross. To verify whether interruption would affect task performance in pre-interruption trials, a pure 2-back task was used as the baseline condition.

**FIGURE 3:**
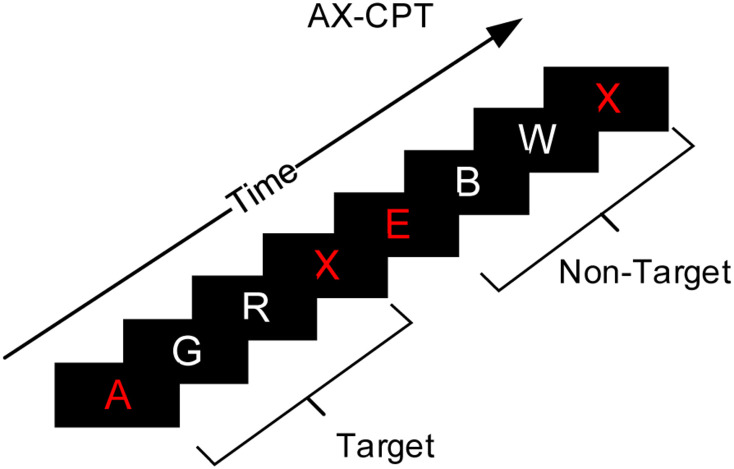
Experiment instruction interface of the AX-CPT paradigm.

Mental fatigue was induced by the AX version of the continuous performance task (AX-CPT), as shown in Figure 3 (van der Linden et al., 2006). The paradigm consisted of one cue stimulus, two random interference stimuli, and one probe stimulus. The stimulus materials were 24 letters, excluding K and Y (which are too similar to X).

### Procedures

This study employed a 2 fatigue state (non-fatigue, fatigue) × 2 task type (IT, ST) × 2 trial type (pre-interruption, post-interruption) within-subject experimental design, and all participants needed to perform three types of tasks. Participants were first trained for 30 mins and given performance feedback to ensure that their performance did not increase significantly during the formal experiment. The formal experiment was divided into fatigue and non-fatigue sessions and carried out in a random order over 2 days. Before the fatigue session, the participants worked on the AX-CPT for 100 mins to induce mental fatigue successfully (Marcora et al., 2009). Participants were required to make judgments of cue-probe sequences. The sequence consisting of cue stimulus A followed by probe stimulus X, with two non-A and non-X random interference stimuli in the middle, was a target. Any other combination was a non-target. The probability of a target trial was 70%, and the probability of a non-target trial was 10%. The participants needed to press the E key in response to target trials and the I key in response to non-target trials. The accuracy of the participants was checked every 30 mins. Only when performance exceeded 85% was fatigue induction considered effective. The Stanford Sleepiness Scale (SSS) (Hoddes et al., 1973) was used to assess each participant’s state before and after the AX-CPT task, and before the non-fatigue session.

For 2-back trials, participants responded by pressing the “f” key with the left hand for a match and the “j” key with the right hand for a mismatch. For math trials, participants needed to judge whether the solution to the math equation was greater than or equal to 50 by pressing the “f” and “j” keys for right and wrong, respectively. Each secondary task consisted of five random math questions in IT. After completing the five math tasks, the 2-back task was resumed. The participants were required to determine whether the current stimulus matched the last two stimuli before the interruption. A suspension with a prolonged presentation of the fixed cross in ST had the same duration as the five math tasks. The two trials following the math tasks or a prolonged fixed cross were regarded as the post-interruption trials for IT and ST, and the other 2-back trials were the pre-interruption trials. There were 2 post-interruption trials and 12 pre-interruption trials between two task interruptions or suspensions. The ratio of match trials and mismatch trials was 1:1, as was the ratio of right to wrong for the math tasks. Each condition contains two blocks with the same processes. There were 156 pre-interruption trials and 24 post-interruption trials in every block of both IT and ST conditions. The baseline consisted of 160 pre-interruption trials in each block.

The experiment sequence adopted the Latin square design. The participants took a short break between the two blocks to ensure no additional fatigue. After completion of each task, the NASA Task Load Index (NASA-TLX) scale was used to evaluate the subjective mental workload.

### Electroencephalography Recording and Preprocessing

A 64-channel Neuroscan SynAmps2 amplifier recorded brain electrical activity with a sampling frequency of 1,000 Hz (Neuroscan Inc., United States). Scalp recordings were referenced online to the electrode between Cz and CPz and re-referenced to the average of the left and right mastoids through offline algebraic computations. The vertical electrooculogram (VEOG) data were recorded from electrodes above and below the left eye. The horizontal electrooculogram (HEOG) data were monitored by placing electrodes at the outer canthi of both eyes. EEG data were collected with all electrode impedances kept below 5 kΩ.

Offline data analyses were conducted using the EEGLAB toolbox of MATLAB R2020a (Hruby and Marsalek, 2002). The continuous EEG signals were filtered using a band-pass filter from 0.1 to 30 Hz. Filtered data were segmented into epochs of -200 to 800 ms after the stimulus and baseline-corrected relative to an interval of -200 to 0 ms for ERP analyses. Eye movement artifacts were removed using independent component analysis (ICA). Trials contaminated with large artifacts (peak to peak deflection exceeding 75 μV) were excluded.

### Data Analysis

We determined time windows centered on the peak by visually inspecting individual data and measured local peak amplitude as defined by Luck (2014). In line with previous studies and the average topography map, P200 was detected as occurring at 180–280 ms from the onset of the stimulus at anterior frontal (F3, Fz, and F4), central (C3, Cz, and C4), and posterior parietal (P3, Pz, and P4) electrodes (Vilà-Balló et al., 2018). Analysis of P300 was confined to the electrodes at anterior frontal (F3, Fz, and F4), central (C3, Cz, and C4), and posterior parietal (P3, Pz, and P4) in the time window of 300–450 ms from the onset of the stimulus (Lin et al., 2020). The theta (4–7 Hz) and alpha bands (8–12 Hz) in the time window of 0–1,000 ms post-stimulus onset were analyzed separately at Fz, Cz, and Pz with baseline window −500 to −200 ms (Boksem et al., 2005). The relative wavelet energies of theta and alpha bands were analyzed using wavelet packet time-frequency analysis (Ting et al., 2008). Daubechies db4 was selected as the mother wavelet because of the high signal-to-noise ratio value (Balasubramanian et al., 2018).

The repeated-measures analyses of variance (rm-ANOVA) were conducted. One-way rm-ANOVAs were used to analyze the effects of task types (IT vs. ST vs. Baseline) on primary task performance under fatigue and non-fatigue conditions. And 2 (fatigue state: fatigue vs. non-fatigue) × 2 (task type: IT vs. ST) × 2 (trial type: pre-interruption vs. post-interruption) rm-ANOVAs were conducted on response times (RT) and accuracy rates (ACC). The three-way rm-ANOVAs were used to analyze the main effects of fatigue state (Fatigue vs. Non-fatigue), task type (IT vs. ST), and trial type (Pre-interruption vs. Post-interruption), as well as their interaction effects on EEG power. For ERP component amplitudes, the 2 (fatigue state: fatigue vs. non-fatigue) × 3 (task type: IT vs. ST vs. Baseline) rm-ANOVAs were firstly conducted to analyze the effect of interruption in pre-interruption trials. Then we conducted three separate 2 (fatigue state: fatigue vs. non-fatigue) × 2 (task type: IT vs. ST) × 2 (trial type: pre-interruption vs. post-interruption) rm-ANOVAs at frontal, central, and parietal electrode sites.

Behavioral data included the RT and ACC for the three types of tasks recorded by E-Prime 3.0 software. Subjective mental workload was assessed using NASA-TLX. One participant’s data were rejected due to excessive eye movement and movement artifacts and low accuracy. The data of the remaining 33 participants were analyzed. The remaining data were statistically analyzed after outlier screening with three standard deviations. An average of 89.91% of trials was accepted for statistical analysis, Table 1 shows the number of trials contributing to the average ERP in each condition. All analyses were carried out using SPSS 25.0. The Greenhouse–Geisser method was used to correct the variance analysis data when sphericity assumptions were violated. A *p* value < 0.05 was considered significant, and partial eta-square $\left(\eta_{p}^{2}\right)$ was reported as a measure of effect size.

**TABLE 1:** Descriptive statistics for the number of trials in each condition (Mean ± SD).

**Task type**	**Interruption task**		**Suspension task**		**Baseline**
**Trial Type**	**Pre-interruption**	**Post-interruption**	**Pre-interruption**	**Post-interruption**	**–**
Non-fatigue	278.12 ± 44.38	42.75 ± 8.54	275.43 ± 45.94	41.14 ± 9.46	279.40 ± 54.74
Fatigue	282.12 ± 36.07	45.24 ± 3.56	292.52 ± 29.13	43.61 ± 5.22	298.55 ± 27.95

## Results

### Behavioral Results

The descriptive statistics of the behavioral data under each condition are summarized in Table 2. The effect of task type on primary task performance was analyzed using a one-way rm-ANOVA. The main effect of task type was not significant on overall RT [*F*(2, 64) = 1.058, *p* = 0.353, $\eta_{p}^{2}$ = 0.032] and overall ACC [*F*(2, 64) = 1.583, *p* = 0.213, $\eta_{p}^{2}$ = 0.046]. Surprisingly, although there was no statistical significance on pre-interruption RT [*F*(2, 64) = 0.238, *p* = 0.789, $\eta_{p}^{2}$ = 0.007] and pre-interruption ACC [*F*(2, 64) = 0.079, *p* = 0.924, $\eta_{p}^{2}$ = 0.002], it can be seen from the descriptive statistics that the performance of IT and ST in pre-interruption trials was higher than that of the baseline. This is further analyzed in the discussion.

**TABLE 2:** Descriptive statistics of behavioral data in various conditions (Mean ± SD).

**Fatigue state**	**Task type**	**Trial type**	**ACC**	**RT (ms)**
Non-fatigue	Interruption task	Pre-interruption	0.94 ± 0.05	635.61 ± 140.75
		Post-interruption	0.88 ± 0.12	761.68 ± 156.20
	Suspension task	Pre-interruption	0.95 ± 0.05	627.82 ± 134.10
		Post-interruption	0.91 ± 0.11	712.32 ± 140.20
	Baseline	–	0.94 ± 0.05	642.60 ± 157.06
Fatigue	Interruption task	Pre-interruption	0.92 ± 0.08	693.24 ± 161.45
		Post-interruption	0.84 ± 0.14	862.30 ± 195.32
	Suspension task	Pre-interruption	0.91 ± 0.09	703.64 ± 164.53
		Post-interruption	0.85 ± 0.15	830.53 ± 195.55
	Baseline	–	0.92 ± 0.07	699.45 ± 162.12

**FIGURE 4:**
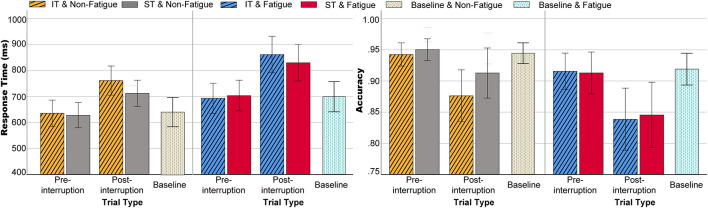
Behavioral data including RT (left) and ACC (right) in various conditions. Error bars indicate 95% confidence intervals (CIs).

The three-way rm-ANOVAs were performed with fatigue state, task type, and trial type as the three within-subject factors. For RT, the main effects of fatigue state [*F*(1, 32) = 14.634, *p* = 0.001, $\eta_{p}^{2}$ = 0.314] and trial type [*F*(1, 32) = 105.690, *p* < 0.001, $\eta_{p}^{2}$ = 0.768] were significant. The fatigue × trial type interaction [*F*(1, 32) = 7.839, *p* = 0.009, $\eta_{p}^{2}$ = 0.197] and the task type × trial type interaction were both significant[*F*(1, 32) = 9.678, *p* = 0.004, $\eta_{p}^{2}$ = 0.232], as shown in Figure 4 (left). Further simple effects analysis showed that the RT of post-interruption trials was longer for IT than for ST [*F*(1, 32) = 8.413, *p* = 0.007, $\eta_{p}^{2}$ = 0.208], and there was no significant difference in the pre-interruption trials [*F*(1, 32) = 0.018, *p* = 0.894, $\eta_{p}^{2}$ = 0.001]. The interaction of the three factors was not significant [*F*(1, 32) = 0.001, *p* = 0.983, $\eta_{p}^{2}$ = 0.001].

The ACC results indicate that the main effects of fatigue [*F*(1, 32) = 12.116, *p* = 0.001, $\eta_{p}^{2}$ = 0.275] and trial type were significant [*F*(1, 32) = 27.083, *p* < 0.001, $\eta_{p}^{2}$ = 0.458]. As shown in Figure 4 (right), both fatigue and interruption reduced accuracy, and accuracy of post-interruption trials was significantly lower in the fatigue state than in the non-fatigue state. The main effect of task type was not significant [*F*(1, 32) = 2.313, *p* = 0.138, $\eta_{p}^{2}$ = 0.067], but accuracy for IT was significantly lower than accuracy for ST in a non-fatigue state [*F*(1, 32) = 8.931, *p* = 0.005, $\eta_{p}^{2}$ = 0.218]. The interaction of the three factors was not significant [*F*(1, 32) = 1.047, *p* = 0.314, $\eta_{p}^{2}$ = 0.032].

### Subjective Assessment

The paired *t*-test was used to analyze the SSS scores before the fatigue and the non-fatigue sessions. The scale score was significantly higher after fatigue induction (*M* = 5.45, SD = 1.13) than before the AX-CPT task (*M* = 2.30, SD = 2.28) [*t*(32) = −18.036, *p* < 0.001], which suggests that AX-CPT successfully induced fatigue.

A two-way rm-ANOVA of 2 (fatigue status: non-fatigue, fatigue) × 3 (task type: task interruption, suspension interruption, non-interruption) was performed on the NASA-TLX scale. The results show significant main effect of task type [*F*(2, 64) = 45.493, *p* < 0.001, $\eta_{p}^{2}$ = 0.587]. The mental load was higher for IT than for ST and for the baseline task. There was no significant interaction between the two factors [*F*(2, 64) = 2.657, *p* = 0.078, $\eta_{p}^{2}$ = 0.077].

### Event-Related Potentials Results

#### P200

For P200 amplitudes in the pre-interruption trials, there was no significant differences among the three types of tasks at frontal [*F*(2, 64) = 1.105, *p* = 0.338, $\eta_{p}^{2}$ = 0.034], central [*F*(2, 64) = 0.384, *p* = 0.682, $\eta_{p}^{2}$ = 0.012], and parietal electrodes [*F*(2, 64) = 0.020, *p* = 0.980, $\eta_{p}^{2}$ = 0.001].

**FIGURE 5:**
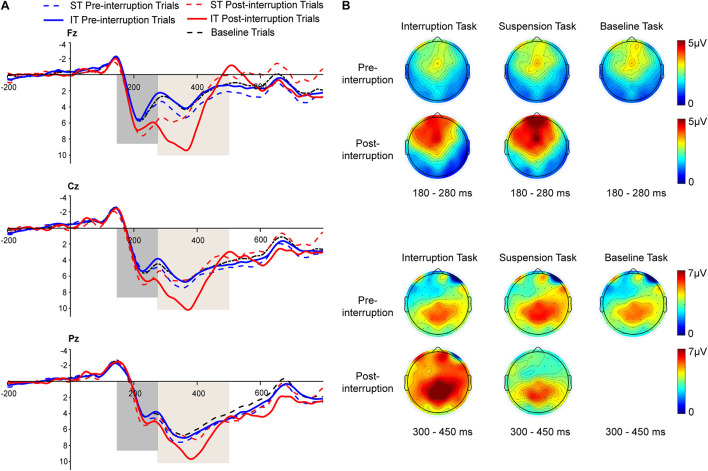
**(A)** Grand averages of P200 and P300 event-related potentials (ERP) components at the Fz, Cz, and Pz electrodes under *non-fatigue* state. The black dashed line represents all trials of the baseline task. The blue and red dashed lines represent the pre- and post-interruption trials of the suspension task (ST). The blue and red solid lines represent the pre- and post-interruption trials of the interruption task (IT). **(B)** The scalp topographies display the mean amplitude under *non-fatigue* state of 180–280 ms for P200 and 300–450 ms for P300.

**FIGURE 6:**
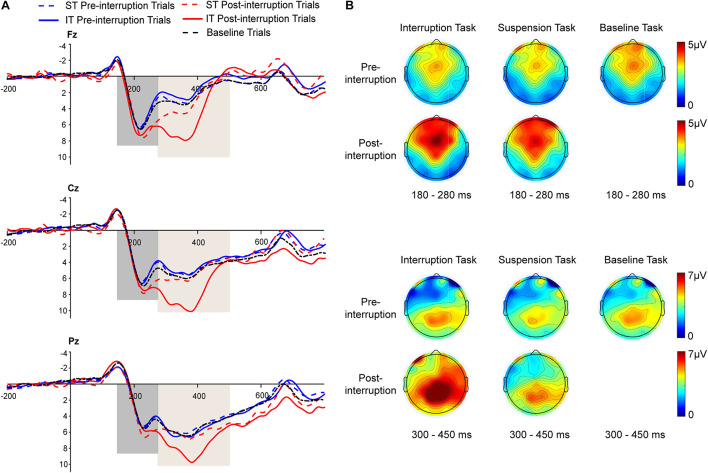
**(A)** Grand averages of P200 and P300 ERP components at the Fz, Cz, and Pz electrodes under *fatigue* state. The black dashed line represents all trials of the baseline task. The blue and red dashed lines represent the pre- and post-interruption trials of the suspension task (ST). The blue and red solid lines represent the pre- and post-interruption trials of the interruption task (IT). **(B)** The scalp topographies display the mean amplitude under *fatigue* state of 180–280 ms for P200 and 300–450 ms for P300.

**FIGURE 7:**
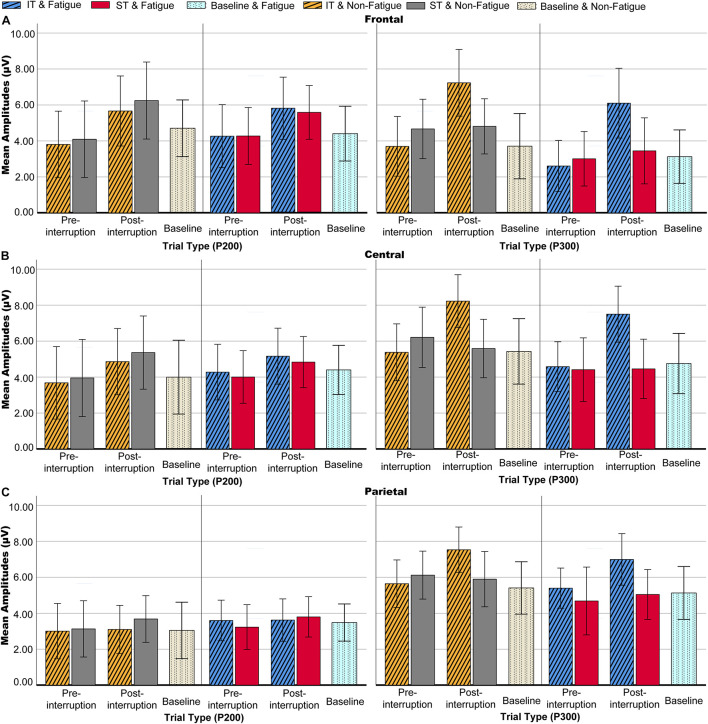
Mean P200 and P300 amplitudes of each condition at electrode sites. **(A)** Frontal electrodes. **(B)** Central electrodes. **(C)** Parietal electrodes. Error bars represent 95% CIs.

Averaged ERPs and topographical maps under non-fatigue and fatigue are shown in Figures 5, 6. The three-way rm-ANOVAs (Table 3) and Bonferroni correction *post hoc* analyses were conducted at three electrode sites. Figure 7 shows the mean P200 amplitudes of each condition at frontal, central, and parietal electrodes. At frontal electrodes, the amplitudes of the post-interruption trials were significantly higher than those of the pre-interruption trials, both in IT condition [*F*(1, 32) = 19.899, *p* < 0.001, $\eta_{p}^{2}$ = 0.571] and ST condition [*F*(1, 32) = 5.517, *p* = 0.025, $\eta_{p}^{2}$ = 0.151]. At central electrodes, the same effect of trial type was only significant in IT condition [*F*(1, 32) = 10.962, *p* = 0.002, $\eta_{p}^{2}$ = 0.255], but not occurred in ST condition [*F*(1, 32) = 3.300, *p* = 0.079, $\eta_{p}^{2}$ = 0.093]. No other main effects or interaction effects were observed.

**TABLE 3:** Three-way rm-ANOVA analyses results for P200 and P300 amplitudes at frontal, central, and parietal electrodes.

**Frontal**	**Mean P200 amplitude**			**Mean P300 amplitude**		
**Effect**	***F***	***p***	** $\eta_{p}^{2}$ **	***F***	***p***	** $\eta_{p}^{2}$ **
Fatigue	0.001	0.997	<0.001	0.854	0.362	0.027
Task Type	0.184	0.671	0.006	11.389	0.002**	0.269
Trial Type	18.137	<0.001***	0.370	5.593	0.024*	0.153
Fatigue × Task Type	2.294	0.140	0.069	2.119	0.156	0.064
Fatigue × Trial Type	1.117	0.299	0.015	0.637	0.431	0.020
Task Type × Trial Type	2.074	0.160	0.063	39.402	<0.001***	0.560
Three factors interaction	0.621	0.148	0.020	2.198	0.148	0.066

**Central**	**Mean P200 amplitude**			**Mean P300 amplitude**		
**Effect**	***F***	***p***	** $\eta_{p}^{2}$ **	***F***	***p***	** $\eta_{p}^{2}$ **

Fatigue	0.452	0.506	0.014	0.970	0.332	0.029
Task Type	0.003	0.954	<0.001	21.021	<0.001***	0.396
Trial Type	10.124	0.003**	0.240	1.614	0.213	0.048
Fatigue × Task Type	0.091	0.765	0.003	0.539	0.468	0.017
Fatigue × Trial Type	2.974	0.094	0.085	1.328	0.258	0.040
Task Type × Trial Type	0.217	0.645	0.007	39.886	<0.001***	0.555
Three factors interaction	0.052	0.820	0.002	4.988	0.033*	0.135

**Parietal**	**Mean P200 amplitude**			**Mean P300 amplitude**		
**Effect**	***F***	***p***	** $\eta_{p}^{2}$ **	***F***	***p***	** $\eta_{p}^{2}$ **

Fatigue	0.231	0.634	0.007	1.106	0.301	0.033
Task Type	0.001	0.981	<0.001	8.998	0.005**	0.219
Trial Type	0.011	0.916	<0.001	0.941	0.339	0.029
Fatigue × Task Type	0.583	0.451	0.018	0.040	0.843	0.001
Fatigue × Trial Type	0.902	0.349	0.027	0.265	0.610	0.008
Task Type × Trial Type	0.019	0.892	0.001	15.404	<0.001***	0.324
Three factors interaction	1.992	0.168	0.059	3.893	0.057	0.108

***p < 0.001; **p < 0.01; and *p < 0.05.

#### P300

In the pre-interruption trials, there was no significant difference in the mean P300 amplitude among the three task types at central [*F*(2, 64) = 2.079, *p* = 0.133, $\eta_{p}^{2}$ = 0.061] and parietal electrodes [*F*(2, 64) = 0.020, *p* = 0.274, $\eta_{p}^{2}$ = 0.040]. At frontal electrodes, the mean P300 amplitudes of pre-interruption trials in ST condition was higher than in IT condition [*F*(2, 64) = 3.450, *p* = 0.038, $\eta_{p}^{2}$ = 0.100].

Averaged ERPs and topographical maps under non-fatigue and fatigue are shown in Figures 5 and 6. Figure 7 shows the mean P300 amplitudes of each condition at frontal, central, and parietal electrodes and Table 3 shows the statistic results. At frontal electrodes, the main effects of task type and trial type were significant. There was a significant interaction between task type and trial type. The P300 amplitudes were higher for IT than for ST, especially in post-interruption trials [*F*(1, 32) = 25.746, *p* < 0.001, $\eta_{p}^{2}$ = 0.454]. And the amplitude in post-interruption trials was higher than in pre-interruption trials. Further simple effects analysis shows that the amplitude difference by trial type was noted only in IT condition [*F*(1, 32) = 42.531, *p* < 0.001, $\eta_{p}^{2}$ = 0.571], but not in ST condition [*F*(1, 32) = 0.968, *p* = 0.333, $\eta_{p}^{2}$ = 0.030].

At central electrodes, there were significant main effects of the task type and significant task type × trial type interaction effect. And the three factors interaction effect was significant. Follow-up analyses indicated that task interruption elicited a larger P300 in post-interruption trials than pre-interruption trials in IT condition, [*F*(1, 32) = 33.404, *p* < 0.001, $\eta_{p}^{2}$ = 0.511], but not in ST condition [*F*(1, 32) = 3.826, *p* = 0.059, $\eta_{p}^{2}$ = 0.107]. The interaction effect of fatigue and trial type was significant in ST condition [*F*(1, 32) = 5.277, *p* = 0.028, $\eta_{p}^{2}$ = 0.412], but not in IT condition [*F*(1, 32) = 0.182, *p* = 0.673, $\eta_{p}^{2}$ = 0.006]. Further simple effects analysis showed that the P300 amplitudes of pre-interruption trials were higher than post-interruption trials in IT under non-fatigue state [*F*(1, 32) = 6.172, *p* = 0.018, $\eta_{p}^{2}$ = 0.162], but there was no significant different under fatigue[*F*(1, 32) = 0.975, *p* = 0.331, $\eta_{p}^{2}$ = 0.030].

At parietal electrodes, we detected significant main effects of the trial type and a significant task type × trial type interaction effect. In IT condition, task interruption elicited a larger P300 in post-interruption trials than pre-interruption trials [*F*(1, 32) = 12.584, *p* = 0.001, $\eta_{p}^{2}$ = 0.282], but not in ST condition [*F*(1, 32) = 1.723, *p* = 0.199, $\eta_{p}^{2}$ = 0.051].

### Electroencephalography Results

#### Theta Band Power

**FIGURE 8:**
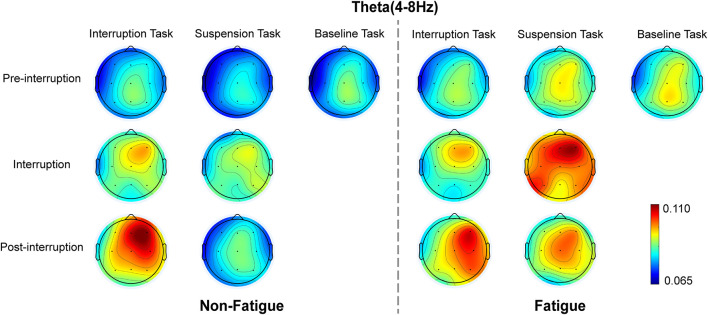
Topographic representation of the grand average of the relative theta energy in various conditions.

**FIGURE 9:**
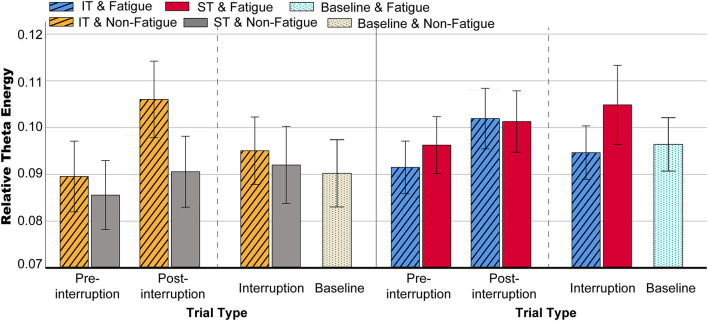
Mean relative theta energy in various conditions. Error bars indicate 95% CIs.

The mean relative theta energies at electrodes Fz, Cz, and Pz were calculated for statistical analysis of variance. The results indicate that theta band power did not vary with task type in pre-interruption trials [*F*(2, 64) = 1.300, *p* = 0.275, $\eta_{p}^{2}$ = 0.013]. Figure 8 presents the scalp topographies of the relative theta energy. The theta activity was typically centered over the frontal and central regions, especially in post-interruption trials under fatigue. The three-way rm-ANOVA for the theta power (Table 4) revealed the main effects of task type and trial type. The theta power was significantly higher during IT than during ST, and the theta activity was higher in post-interruption trials than in pre-interruption trials. We found an interaction effect between fatigue and task type. Subsequent analyses show that the higher theta power for IT than for ST was significant only in the non-fatigue state [*F*(1, 32) = 23.669, *p* < 0.001, $\eta_{p}^{2}$ = 0.195], whereas no such difference was evident in the fatigue state [*F*(1, 32) = 0.862, *p* = 0.355, $\eta_{p}^{2}$ = 0.009 (see Figure 9).

**TABLE 4:** The 2 (Fatigue state: non-fatigue, fatigue) × 2 (Task Type: IT, ST) × 2 (Trial Type: pre-interruption, post-interruption) rm-ANOVA analyses results for theta and alpha power.

	**Theta**			**Alpha**		
**Effect**	***F***	***p***	** $\eta_{p}^{2}$ **	***F***	***p***	** $\eta_{p}^{2}$ **
Fatigue	2.678	0.105	0.027	8.191	0.005**	0.077
Task Type	6.903	0.010*	0.066	1.692	0.196	0.017
Trial Type	62.192	<0.001***	0.388	1.764	0.187	0.018
Fatigue × Task Type	14.854	<0.001***	0.132	8.104	0.005**	0.076
Fatigue × Trial Type	0.880	0.351	0.009	3.481	0.065	0.034
Task Type × Trial Type	14.479	<0.001***	0.129	9.092	0.003**	0.085
Three factors interaction	1.462	0.229	0.015	5.027	0.027**	0.049

***p < 0.001; **p < 0.01; and *p < 0.05.

We further analyzed the effects of interruption trials on theta power, including math tasks in IT and fixation suspension in ST. The interaction effect between task type and trial type was significant [*F*(2, 64) = 12.655, *p* < 0.001, $\eta_{p}^{2}$ = 0.114]. The simple effects show that the interruption trials of ST were higher than pre-interruption trials (*p* = 0.005) [*F*(2, 64) = 6.230, *p* = 0.004, $\eta_{p}^{2}$ = 0.060]. In IT condition, the interruption trials were significantly lower than post-interruption trials (*p* < 0.001) [*F*(2, 64) = 30.311, *p* < 0.001, $\eta_{p}^{2}$ = 0.236].

#### Alpha Band Power

**FIGURE 10:**
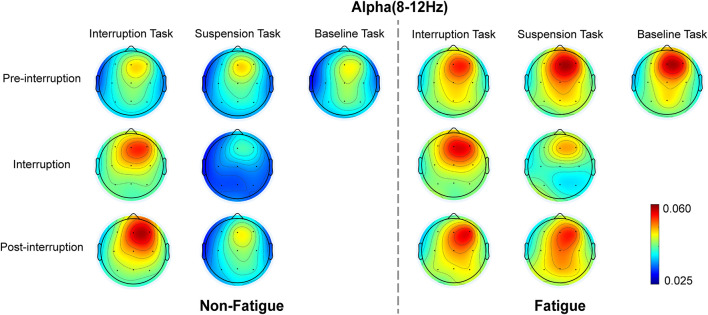
Topographic representation of the grand average of the relative alpha energy in various conditions.

**FIGURE 11:**
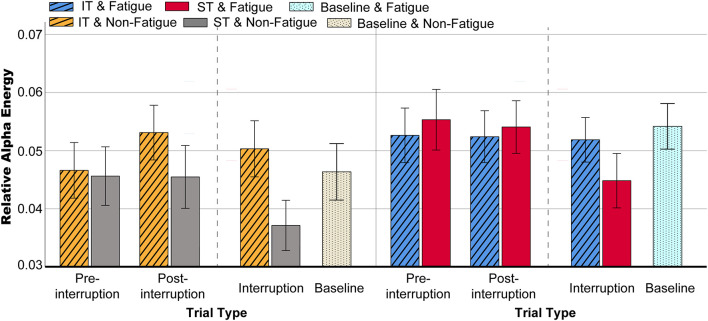
Mean relative alpha energy in various conditions. Error bars indicate 95% CIs.

The scalp topographies of the relative alpha energy show that the alpha oscillation is mainly localized over the frontal and central electrodes and across the frontal and posterior areas in post-interruption trials under fatigue state (Figure 10). The results from the oscillatory alpha power analysis are displayed in Figure 11. The results show that there was no significant main effect of task type during pre-interruption trials [*F*(2, 64) = 0.243, *p* = 0.785, $\eta_{p}^{2}$ = 0.002]. The results of the three-way rm-ANOVA (Table 4) show a significant main effect of fatigue and a three-way interaction effect. The following analyses indicate that the two-way interaction between fatigue and task type was significant for post-interruption trials [*F*(1, 32) = 11.307, *p* = 0.001, $\eta_{p}^{2}$ = 0.103], but not significant for pre-interruption trials [*F*(1, 32) = 2.301, *p* = 0.132, $\eta_{p}^{2}$ = 0.023]. Further simple-effect analyses show that the post-interruption trials of IT increased alpha power in the non-fatigue state compared to ST [*F*(1, 32) = 24.626, *p* < 0.001, $\eta_{p}^{2}$ = 0.201]. However, this difference in task types did not found under fatigue [*F*(1, 32) = 0.828, *p* = 0.365, $\eta_{p}^{2}$ = 0.008].

The subsequent analyses of the effects of interruption trials indicate that the interaction effect between task type and trial type was significant [*F*(2, 64) = 31.332, *p* < 0.001, $\eta_{p}^{2}$ = 0.242]. Alpha activity in the interruption trials was significantly lower than in the pre- (*p* < 0.001) and post- (*p* < 0.001) interruption trials in ST [*F*(2, 64) = 26.596, *p* < 0.001, $\eta_{p}^{2}$ = 0.213]. In IT condition, alpha power of interruption trials was not significantly different from pre- (*p* = 0.420) and post- (*p* = 0.622) interruption trials.

## Discussion

This study has investigated the effects of interruption on WM and the moderating effect of fatigue from behavioral and neuroscience perspectives. Using the 2-back paradigm, we examined behavioral and EEG data differences during task and suspension interruptions in non-fatigue and fatigue states. The results support our hypotheses. On the behavioral level, we found that interruptions reduced ACC and increased RT of post-interruption trials. The interruptions caused by a secondary task had a more strongly negative effect on performance than suspension interruption in the non-fatigue state. Interruption had a greater disruption to performance in the fatigue state than in the non-fatigue state. The theta power for IT improved significantly after interruption; the alpha power was significantly reduced in the interruption trials. ERP analyses show that the amplitudes of P200 and P300 increased significantly after the interruption, which reflects the effect of interruption on attention and WM (Lenartowicz et al., 2010). The significant increase of alpha power and P300 amplitude after task interruption suggests inhibition control to irrelevant information from secondary task interruption (Polich, 2007; Clayton et al., 2015). The changes of EEG and ERP indexes in different mental fatigue states show that fatigue negatively moderates performance and further aggravates the negative effect of interruption on WM and primary performance.

### Behavioral Measures

Secondary task interruption and suspension interruption decrease performance in post-interruption trials to varying degrees. However, compared with the baseline task without interruption, the total performance of the two types of interruption tasks (IT and ST) showed no significant decline. The result is unexpected. A possible explanation is the speedup effect following an interruption, and it is reset by a new interruption (Trafton and Monk, 2007). Ratwani et al. (2006) thought the speeding up after interruption can be attributed to increased perceptual speed (faster fixation) rather than increased speed in the cognitive system. We are more inclined to the compensatory mechanism of cognitive control. The compensatory control model proposed by Hockey (1997) also assumes that the behavior is goal-oriented and achieves resource allocation through an effort monitor. After an interruption, the active control of participants, including increased working memory or executive control, improved performance for a short time. Speier et al. (1999) confirmed that interruption promotes behavior performance briefly when performing simple or repetitive tasks; arousal or stress is elevated when interruptions occur, and increased arousal leads to more attention being given to subsequent tasks, resulting in better performance (Speier et al., 1999). In the present study, behavioral performance declined more after interruptions in the fatigue state. Thus, even if the speeding-up effect improved performance on the pre-interruption trials slightly (see Table 2), it did not significantly improve the overall performance. This is because of the side-effect of compensatory cost. The speedup effect requires greater effort to maintain performance, which increases subjective mental workload (Fonseca et al., 2018). It can be seen from the NASA-TLX results that IT caused the highest subjective mental load, followed by ST, and the non-interrupted baseline task had the lowest load. Furthermore, in a state of fatigue, people tend to choose activities that require less effort or less use of high-level control actions (Hockey, 1997).

### Electroencephalography Power

As expected, theta power was enhanced in the post-interruption trials of IT. This is consistent with previous studies, where theta power has been shown to increase with memory load (Jensen and Tesche, 2002; Lei and Roetting, 2011). It has been demonstrated that the theta band plays an active role in WM control (Onton et al., 2005) and that theta power is higher as encoded information increases (Jensen and Tesche, 2002), especially in information encoding and retention (Sauseng et al., 2010). In the present study, during interruption of IT, information maintenance was required. The post-interruption trials involved both information activation and a new 2-back trial encoding. Thus, theta power gradually increased during the entire interruption process. Moreover, in the ST interruption trials, the theta power was not significantly lower than post-interruption as the participant kept repeating and recalling during the suspension, which lends further support to this statement.

A meta-analysis of the influence of mental fatigue on brain activity reported that theta activity increased with mental fatigue (Tran et al., 2020). In the present study, the theta power of ST increased in the fatigue state. This result is consistent with previous findings. However, the increase of theta power was not statistically significant in IT condition. This may be due to the different task types used; the analysis of Tran et al. (2020) is based on driving tasks rather than working memory tasks. The possible explanation for these results is that mental fatigue damages attention and memory processing (Hope, 2016). There was less speeding up after the interruption for compensating actively for the negative effects of interruption than non-fatigue state even if the participants had put in more effort. The increased theta activity is related to item encoding in WM (Proskovec et al., 2019), which indicates that the memory items effectively processed in fatigue state are limited, resulting in a decline in ACC. In ST condition, the participants had low memory demand, theta power in the fatigue state was higher than that in the non-fatigue state.

Previous studies have found that alpha oscillations are closely related to WM (Clayton et al., 2015). Unlike theta oscillations, alpha oscillations have always been associated with the inhibition of task-irrelevant information (Manza et al., 2014). The present results are consistent with the claim that greater alpha activity indicates an increase in cortical inhibition, reflected in the large difference between ST and IT in the interruption trials. It was necessary to inhibit interference information from the math tasks during IT but not during ST. Consequently, alpha power was reduced in ST.

The moderating effect of fatigue state on alpha power was similar to that for the theta band. Compared to large increases in theta activity, there were moderate to large increases in alpha activity during mental fatigue (Tran et al., 2020). The enhanced alpha power caused by fatigue was reflected in the trial of ST only. For IT, in the fatigue state, there was a reduction in inhibitory alpha power in task-relevant cortical areas (Mazaheri et al., 2009), which led to a decrease, not an increase, in alpha power.

### Event-Related Potentials Components

From the mean P200 amplitude perspective, the difference in amplitude between trial types implies a change of attention in stimulus switching (Luck, 2014; Stefanics et al., 2014). The current study reports increased P200 amplitude in the post-interruption trials compared to the pre-interruption trials. In general, an increase in the participant’s attention may facilitate perceptual processing in visual search, particularly for encoding (Dunn et al., 1998), resulting in a decrease in the amplitude of P200 (Crowley and Colrain, 2004). A greater P200 amplitude is associated with reduced attentional resources to the primary tasks. ST made the original workflow pause completely in a short time, resulting in a release of more attentional resources. In addition, P200 is related to the WM process (Lefebvre et al., 2005), and P200 amplitude reflects early short-term memory storage (Chapman et al., 2007) and memory activation, which depends on the PFC. Whether there was interference or not, the activity of PFC was observed before retrieval (Sakai, 2003). This is also consistent with the larger P2 amplitude of PFC after task and suspension interruptions.

As a typical indicator of the WM mechanism, P300 amplitude is also sensitive to attentional resources and relates to information interference control, memory maintenance, and updating (Singh et al., 2018). Regardless of fatigue or non-fatigue state, the amplitudes of P300 in the post-interruption trials were significantly higher than in the pre-interruption trials, consistent with previous experimental studies, which found an n-back lure and workload effect (Vilà-Balló et al., 2018; Zickerick et al., 2020b). The P300 component is correlated with rapid neural inhibition of continuous activity and promotes the transmission of information from the frontal lobe to the temporal-parietal lobe (Polich, 2007). The inhibition hypothesis has been confirmed in several experiments (Smith et al., 2006, 2013), and the P300 amplitude increased with the enhancement of brain inhibition (Randall and Smith, 2011). Our results indicate that participants gave more attentional resources to suppressing interference information from task interruption, leading to a significant increase in the P300 amplitude. In ST, where there was no additional interference information unrelated to the primary task, no significant difference in amplitude was found.

The decreased P300 amplitudes after fatigue show that mental fatigue is detrimental to the attention and memory processing of the brain (Zhao et al., 2012). Compared with the non-fatigue state, in the fatigue state the P300 amplitude decreased to different degrees under various conditions, which indicates that there was little attentional resource engagement under fatigue and that the overall effort on information processing decreased.

There was a significant difference in P300 amplitude between IT and ST in the fatigue state, but no such difference was found in the non-fatigue state. This illustrates the moderating effect of fatigue on WM following interruption. Given the increased information processing demand in IT under fatigue, it was necessary to allocate more attentional resources to enhancing the inhibitory function, resulting in greater mental effort (Kok, 2001). This means that the P300 amplitude was not significantly reduced, even under fatigue. However, as IT did not demand inhibitory control for irrelevant information, the post-interruption trials were mainly affected by fatigue, and the P300 amplitude decreased. In line with the behavioral data, the speeding-up effect observed in the non-fatigue state was reflected in a slight increase in P300 amplitude for IT and ST in the pre-interruption trials.

The results of the topographic map show that the prefrontal-parietal network played a critical role in the interruption process, and previous research has found it to be closely connected to attention and WM processes (Cabeza and Nyberg, 2000; Duncan, 2006). As attention shifts and the amount of information in WM increases, the activity of the prefrontal-parietal network also increases, which is in line with the experimental results of this study. The changes in the EEG bands and ERP components reflect the processes of central executive control. The different stimuli trigger top-down attention-switching during the interruption, as shown here for P200. Theta power reflects the WM encoding and maintenance. The alpha oscillations and P300 are more closely related to irrelevant information inhibition and WM updating (Wongupparaj et al., 2018). When the interrupted primary task is restored, attentional relocation and goal reactivation usually lead to a decline in speed and accuracy (Couffe and Michael, 2017). The decrease of brain activity after fatigue indicates the impairment of the WM process, especially the PFC related to memory reactivation after interruption.

Our results provide a better understanding of the cognitive mechanism of performance decline through neurophysiological data and support for the MFG theory (Altmann and Trafton, 2002). Task interruption required the participants to allocate attentional resources to secondary tasks. The new goals for the secondary task were encoded, which impaired the WM for the primary task, and the old goals (the WM of the primary task) decayed gradually over time. After an interruption, it was necessary to inhibit the irrelevant information for the secondary task and to refocus attention on the primary task, reactivating the old goals. This led to a resumption lag after interruption. For simple primary tasks, additional attention resources can be used to prepare for subsequent task-related stimuli. This supports studies that believe a simple IT positively affects performance (Speier et al., 2003).

Further, the current results reveal that the effective suppression of interference information is the key to determining WM performance after interruption. The P300 amplitude and alpha energy increase during the interruption task, while not occur in the suspension task, illustrate that in addition to the decay of WM over time, the interference of irrelevant information exerted a stronger effect on WM performance. Mental fatigue further exacerbated the negative effect of interruption on WM. Even if the participants made efforts to compensate for the performance decline caused by interruption, there was no significant overall impact on performance. Thus, interruption, especially caused by secondary tasks under fatigue, had a greater negative effect on WM and related work performance. The impairing effects of mental fatigue on brain suppression result in more destructive interruptions that occur under fatigue conditions. Therefore, we propose that effective top-down suppression of irrelevant information is a mechanism of improved WM performance after interruptions.

### Limitations and Future Directions

This research has several limitations. First, the sample size used in the study is relatively small. Then, the primary task used in our experiment was a simple spatial 2-back task, which can be performed with the facility after practice. When the execution demand was low, there were more resources available to maintain relevant information. The participants had sufficient attention resources to deal with the interruption, generating a speeding-up effect (Cellier and Eyrolle, 1992). However, many human–computer interaction tasks are much more complicated than the 2-back paradigm. This study is only a preliminary examination of the psychophysiological mechanism of the interruption process and the moderating effect of fatigue. Several questions remain to be answered, particularly for human–computer interaction tasks, whether the emergence of interruption produces a speeding-up effect and whether the positive effect of interruption continues.

## Conclusion and Implications

This study increases understanding of the neural mechanism of cognitive activity during the interruption and the moderating effect of fatigue on interruption. Most notably, both task interruption and suspension interruption have negative effects on WM after interruption. Increased P200 and P300 amplitudes and theta power are associated with impaired attention and the interference of interruption information. The second major finding is that mental fatigue exacerbates the negative effects of interruption on WM and related behavioral performance due to the detriment of cognitive functions. The results show that ERP components of P200 and P300 and alpha and theta waves could be used as physiological indicators to explore the effects of interruption, providing further support for the MFG theory of task interruption.

This study confirmed the negative effects of task interruption on WM based on behavioral results and explained the cognitive mechanism by analyzing the electrophysiological activity of the brain. This initial experimental study provides theoretical support for interruption management and helps to improve the performance of interrupted WM-related tasks. For example, our findings demonstrate that inhibition of irrelevant information affects memory performance after interruption. Dynamic lighting improves fatigue through increased alertness (Shi et al., 2011) to prevent cognitive impairment. Providing more informative peripheral visual cues can enhance activation of related memory to facilitate task performance (Trafton et al., 2003).

## Data Availability Statement

The original contributions presented in the study are included in the article/supplementary material, further inquiries can be directed to the corresponding author.

## Ethics Statement

The studies involving human participants were reviewed and approved by Human Factors and Ergonomics Laboratory, Beijing Jiaotong University. The patients/participants provided their written informed consent to participate in this study.

## Author Contributions

YC designed the experiment, analyzed the data, and wrote the original draft. WF supervised the process and proofread the manuscript. BG reviewed and edited the manuscript and acquired funding. HB assisted with data collection and visualized the data. All authors contributed to the article and approved the submitted version.

## Conflict of Interest

The authors declare that the research was conducted in the absence of any commercial or financial relationships that could be construed as a potential conflict of interest.

## Publisher’s Note

All claims expressed in this article are solely those of the authors and do not necessarily represent those of their affiliated organizations, or those of the publisher, the editors and the reviewers. Any product that may be evaluated in this article, or claim that may be made by its manufacturer, is not guaranteed or endorsed by the publisher.
